# Pharmacological topics of interest for young people: misinformation on TikTok is common 

**DOI:** 10.1007/s00210-025-04518-9

**Published:** 2025-11-14

**Authors:** Alexa Ayana Haß, Roland Seifert

**Affiliations:** Institute of Pharmacology, Hannover Medical School, Carl-Neuberg-Str. 1, D-30655 Hannover, Germany

**Keywords:** TikTok, Social media, Public health, Influencers, Drugs, Birth control, Sex hormones

## Abstract

TikTok is a popular social media platform for young people. This paper aims to investigate the quality of pharmacological content of interest to young people on TikTok. 121 TikTok posts were analysed. The main parameters chosen were presence of misinformation, number of likes/views/comments, advertisement, occupational group, and source of reference. Sex hormones, opioids, and illicit drugs were the most popular topics. Eleven (9%) of 121 posts contained an advertisement. There was significantly more misinformation present in these videos than in posts without advertising. Most posts displayed no source of reference for the given claims (76%). The largest occupational group were influencers with 53 of 121 created videos. Pharmacological education was scarce among content generators. Thirty-two percent of posts contained misinformation, with influencers contributing overproportionally to misinformation. TikTok covers many pharmacological topics of interest to young people. Unfortunately, poor or no referencing of sources and misinformation are common. Content generators often lack proper pharmacological qualification. There is a need for a closer analysis of other social media regarding pharmacological topics. Especially children are estimated to be highly influenced by the portrayal of pharmacological content on social media platforms resulting in possible danger for their health. Protective guidelines for using TikTok and global awareness must be established. Conversely, TikTok must be actively used by official institutions to disseminate correct pharmacological information.

## Introduction

Social media has become an increasing market in our society and has a large influence on people all around the world (Powell and Pring 2024). Since social media content is largely unregulated, it is essential to investigate its effects on public health matters (Powell and Pring 2024). Recent studies show that social media is the most important health resource for young people (McCashin and Murphy 2023). The key role of influencers in health matters was already observed in the Covid-19 pandemic. Influencers promoting hygiene habits like hand disinfection led to positive changes in overall health behaviour (Powell and Pring 2024).

While some studies showed these positive effects, others have found negative effects of influencer marketing on social media especially for young people (Powell and Pring 2024). Advertising healthy foods had no effect on children’s behaviour and promoting unhealthy food choices was linked to an increased energy intake (Powell and Pring 2024). Highly idealised body images created by influencers are proven to have negative effects on body image and self-esteem (Powell and Pring 2024).

Searching for health information on the Internet has been increasingly observed, especially in younger people. A study showed that 92% of participants had used the Internet at least once in the last 12 months to find health-related information (Chu et al. 2017). Ninety-seven percent stated that they choose the Internet as their first medium for research considering health information. One reason for the popularity of the Internet and medical information is that users feel it enables them to be more proactive in medical decision-making. Users can find a wide range of information considering health topics. This is underlined by the fact that all participants in the study expressed their wish for clarity and confirmed health issues as a main reason to use the Internet for their research. The four main advantages stated are convenience, the wide range of accessible information, self-awareness, and the emotional support from the online community. The social aspect of community was particularly important for females. Many participants emphasised that doctors did not give them enough time during consultation to ask questions or to talk about issues. Apart from that, patients where traditional medicine “failed” were prone to search for alternative treatments via the Internet (Chu et al. 2017).

TikTok is an originally Chinese App existing since 2016 (Pedrouzo and Krynski 2023). It has gained a large number of users especially, while the Covid-19 pandemic and is now the most rapidly expanding platform among the youth (McCashin and Murphy 2023). Data collected in the USA suggests that 32.5% of TikTok users are 10–19 years old (McCashin and Murphy 2023). It is estimated that globally, the majority of TikTok users are pre-teens (McCashin and Murphy 2023). TikTok allows use of the app for people aged 13 or older (Montag et al. 2021). Sending direct messages is only allowed for people who are 16 or older (Montag et al. 2021).

TikTok is one of the most addicting social media platforms because of its highly personalised algorithm that collects information about the viewer in a shorter time period than any other social media app (Pedrouzo and Krynski 2023). With the assistance of artificial intelligence and a variety of device permissions required, it can show specific content and advertising based on the user’s activity on several apps (Pedrouzo and Krynski 2023). The continuous stimulation of the dopaminergic system leads to excessive use especially in children (Pedrouzo and Krynski 2023).

The focus of studies published concerning this topic has been food intake and body image (Powell and Pring 2024). Since this is only a small range of topics, our goal was to extend this research from a pharmacological perspective. The effects of TikTok content about popular illicit drugs and sex-related medication have been investigated and are presented in the following. To our knowledge, our study is the first of its kind to analyse pharmacological content on TikTok. Due to the rising attention and importance of the topic, there is great potential for research in this area, and the present study has the character of a pilot study.

## Materials and methods

For this paper, we analysed videos on the social media platform TikTok. Every video analysed was publicly accessible. The search function on TikTok was used to find the videos and afterwards filtered for the highest number of Likes. The use of popularity filters was chosen intentionally to reflect the highest influence and relevancy for users. This aligns with our pilot study’s focus on public health and adolescence. Hashtags that were used in the search process are as follows: “birth control”, “birth control pill”, “estrogen”, “morning after pill”, “planb”, “Viagra”, “sildenafil”, “testosterone”, “anabolic”, “steroids”, “opioids”, “opioid crisis” “drugs”, “party drugs”, “drug awareness”, “cannabinoid”, “weed”, “cbd”, “cbd-oil”, “thc”, “ecstasy”, “molly”, “lsd”, “acid”, “fentanyl awareness”. Because of community guidelines, it is not possible to search for “cannabis” or “fentanyl”.

The final sample contained 121 videos in German and English. All data were documented and analysed in an Excel table. The videos and screenshots of the Number of views were downloaded and archived. The following 13 parameters were analysed: number of likes in absolute numbers, number of Likes in relative numbers, number of comments in absolute numbers, number of comments in relative numbers, number of views in absolute numbers, occupational group of the creator, video length, advertisement, drug/active ingredient mentioned, source reference, presence of incorrect information, animation and language. The language was analysed in terms of the presence of medical terms versus common terms and the language in which the post was created. The initial classification of misinformation was performed by established medical literature. To evaluate the presence of incorrect information, the content was analysed by comparing the claims made with information on PubMed, in medical literature, or other reliable sources like medical journals. Only pharmacological and medical-related claims were analysed. Statistical testing and linear regression were performed with GraphPad Prism (https://www.graphpad.com/features). For statistical testing, the Chi^2^ test and Fisher’s exact test were applied. Linear regression graphics were also designed in GraphPad Prism.

To categorize the profession of each creator, we generated 8 different subcategories. These categories were as follows: doctors, pharmacists, news channels, podcasts, influencers, other expertise, unknown/no expertise, and scientists. To be categorised as an influencer, a creator had to have at least a follower crowd of 100,000 people and/or advertisements. When a creator did not fit the mentioned criteria for an influencer or any of the other professions, he or she was categorised as unknown/no expertise. Some creators were assigned several subcategories, for example a doctor spoke about cannabis in a podcast.

**Fig. 1:**
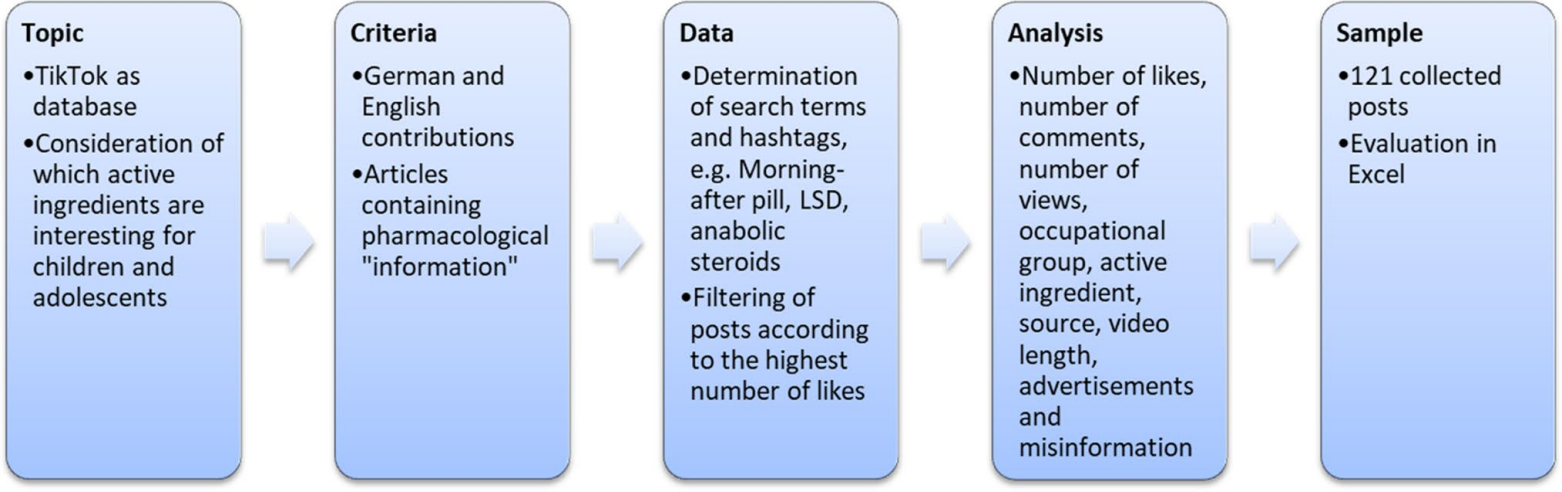
Flowchart describing the process of data collection

The parameter source reference was subcategorised into one source reference, several source references, and no source reference. Figure 1 provides an overview of the methodological procedure. Table 1 provides a summary of important term definitions. Table 2 provides an overview of the general characteristics of the TikTok items analysed. Table 3 provides a code for the entries shown in Table 2.

**Table 1:** Important term definitions

Term	Definition	Citations
Occupational group “influencer”	A creator with ≥ 100,000 followers and/or product promotion	- TikTok influencers are people who have a considerable number of followers and let them participate in their lives. Many have cooperations with brands and promote their products accordingly or post discount codes (https://www.marketinginstitut.biz/blog/tiktok-influencer/#dim-h2-nr4) - Makro-Influencers have over 100,000 followers (Sesar et al. 2022)
Occupational group “unknown/no expertise”	No verified medical, pharmacological, or other scientific qualification	
Occupational group “other expertise”	Counseling centers, non-profit associations/awareness programs, physician assistant	The involvement of nonprofit organizations has been observed to be integral to public health promotion initiatives (Vedel et al. 2020)
Occupational group “news channel”	Media formats with journalistic aspirations	
Misinformation	Contradiction to established scientific literature or clinical guidelines	“Misinformation is false information. Importantly, it is false information that was not created with the intention of hurting others. Misinformation is often started by someone who genuinely wants to understand a topic and cares about keeping other people safe and well. It is then shared by others who feel the same. Everyone believes they are sharing good information – but unfortunately, they are not. And depending on what is being shared, the misinformation can turn out to be quite harmful.” (https://www.who.int/news-room/spotlight/let-s-flatten-the-infodemic-curve)
Advertisement	Explicit marketing for drugs, supplements, or otherwise pharmacologically related products	Social media can be utilised as part of advertising and promotion strategies to communicate incentives such as discounts, special offers, and product-related benefits (Radu et al. 2017)

**Table 2:** Overview on the analysed TikTok content

Number of video files	Number of likes	Number of comments	Occupational group	Number of views	Advertisement	Ingredient	Source	Misinformation	Language
1	33,800	252	1	1,200,000	2	9	2	2	1
2	49,500	892	5	711,100	2	6, 10, 7, 8	3	1	1
3	19,000	167	7	217,500	2	6, 5, 11, 12, 13	3	2	1
4	488,200	3965	1	4,900,000	2	6, 11, 12, 13	3	2	1
5	64,700	1687	1	722,300	2	12, 15, 16, 11	3	1	1
6	32,400	1156	7	397,800	2	7, 10, 6, 17	1	2	1
7	6676	189	7	339,100	2	11	1	2	1
8	13,700	1444	6	421,900	2	11	3	2	1
9	34,300	452	5	782,300	2	6, 18	3	1	1
10	16,100	842	6	645,700	1	6	3	1	1
11	11,600	506	6	525,100	1	6	3	1	1
12	150,800	1461	1, 5	1,000,000	2	6	3	1	1
13	8571	97	6	160,700	1	6	3	2	1
14	18,072	319	6	395,462	1	6	3	2	1
15	34,000	252	1	277,600	2	6	3	1	1
16	106,700	3282	8	1,800,000	2	6	3	2	1
17	19,300	432	6	196,500	2	6	3	2	2
18	355,700	5673	6, 9	3,800,000	1	6	1	1	1
19	100,900	3017	4	1,100,000	2	6	3	2	2
20	1,300,000	36,300	6	6,200,000	2	6	2	2	1
21	32,200	1684	6	2,100,000	1	6	3	1	1
22	15,400	833	1	352,900	2	6	1	2	1
23	207,700	1070	8	2,300,000	2	6	3	1	1
24	92,700	6640	8	1,800,000	2	6	3	2	1
25	78,800	2907	6	1,600,000	2	6	3	2	1
26	362,400	13,000	6	4,100,000	2	6	1	1	1
27	332,400	8347	1, 5	3,000,000	2	6	3	1	1
28	12,000	511	4	141,800	2	6, 15	1	2	2
29	16,000	365	6	162,400	2	8	3	2	2
30	89,400	3331	1	1,100,000	2	8	3	2	1
31	21,900	359	2	570,300	2	8	3	2	1
32	75,500	1036	7	871,900	2	8	3	2	1
33	209,200	4512	4	2,400,000	2	8	2	2	2
34	171,000	5570	6	1,800,000	2	8	1	2	1
35	70,200	1493	8	496,200	2	8	2	1	1
36	200,000	2151	6	2,100,000	2	8	3	2	1
37	4992	64	4	77,500	2	8	3	1	1
38	36,700	477	4	791,000	2	8	2	2	1
39	30,300	671	8	314,200	2	8	1	1	2
40	2,600,000	27,600	6	14,500,000	2	5, 11, 12	3	1	1
41	266,200	2857	7	2,700,000	2	5, 11, 19	3	2	1
42	102,000	1619	6	2,300,000	2	5	3	2	1
43	31,300	1108	6	576,100	2	5	3	1	1
44	213,700	2885	8	1,900,000	2	5	1	2	1
45	58,400	1476	7	1,300,000	2	5, 12, 13, 14, 16, 19	1	1	1
46	11,681	297	6	323,770	2	5, 11, 19	3	2	1
47	382,500	7152	6	3,400,000	2	5, 19	3	2	1
48	128,800	7046	6	1,900,000	2	5, 11	3	2	1
49	94,500	4479	7	1,300,000	2	5, 20	1	2	1
50	2265	73	6	74,000	2	5, 11	3	2	1
51	538,700	12,300	6	6,700,000	2	5	1	1	1
52	24,900	827	1	438,900	2	5, 15	3	1	1
53	83,800	405	6	1,500,000	2	5	3	2	1
54	57,200	958	7	1,300,000	2	5, 13, 12	1	2	1
55	85,100	725	1	1,500,000	2	5	1	2	1
56	17,100	586	4	1,300,000	2	5, 11	3	2	2
57	10,400	74	1, 7	773,900	2	5	3	2	1
58	10,600	323	1, 7	1,000,000	2	5, 6, 16	3	2	1
59	23,700	1558	1	624,500	2	5, 11	3	2	1
60	242,400	6367	6	2,400,000	1	7, 10	3	2	1
61	1,400,000	14,500	4	10,700,000	2	7	2	2	1
62	10,900	170	2	162,500	2	7, 10	3	1	1
63	79,500	1508	7	523,000	2	7	3	2	1
64	109,600	1166	6	646,300	2	7	1	2	1
65	130,900	1341	6	894,700	2	7,8	3	2	2
66	86,800	2313	1	787,200	2	7	3	2	1
67	233,900	2150	6	1,500,000	2	7	3	1	1
68	183,700	692	6	3,700,000	2	1	3	1	1
69	246,200	960	1	4,300,000	2	1	3	2	1
70	129,600	572	6	1,400,000	2	1	3	2	2
71	142,800	1315	8	1,900,000	2	1	3	2	1
72	15,100	238	6	234,300	2	1	1	2	2
73	217,300	5453	1	2,600,000	2	1	3	2	2
74	70,300	688	6	2,100,000	2	1	1	2	1
75	517,300	6173	6	4,900,000	2	1	3	1	1
76	1,500,000	9036	5, 6	17,000,000	2	1	3	2	1
77	364,900	3293	1	2,800,000	2	1	1	2	1
78	15,500	250	2, 6	420,800	1	1	3	2	1
79	68,200	2255	8	990,900	2	1	3	1	1
80	1034	77	4	54,600	2	1	3	2	1
81	206,000	3572	5, 6	k.A	2	1	3	1	1
82	1,400,000	13,400	8	11,500,000	2	1	3	2	1
83	1,200,000	7385	6, 8	4,300,000	2	1	3	1	1
84	598,200	2518	5, 6	5,000,000	2	1	3	1	1
85	796,100	7488	6	10,700,000	2	1	3	1	1
86	227,600	3498	5, 6	2,000,000	2	1	3	1	1
87	486,600	4631	6	5,000,000	2	1	3	2	1
88	250,600	2165	1	1,400,000	2	1	2	2	1
89	588,700	5820	1, 5, 6	8,100,000	2	1	3	1	1
90	1,100,000	7263	5, 6	13,500,000	2	1	3	2	1
91	139,500	1507	8	1,700,000	2	1	3	2	1
92	184,500	2204	6	2,200,000	2	1	3	1	2
93	175,600	1459	6	1,800,000	2	1	3	2	2
94	201,000	8025	8	4,000,000	2	1	3	1	2
95	542,700	9844	6	4,500,000	2	1	3	1	1
96	63,200	335	2	2,000,000	2	2, 21	3	2	1
97	108,700	1547	1	1,100,000	2	2	3	1	1
98	359,900	3158	1	3,400,000	2	1	3	2	1
99	119,000	655	8	1,600,000	2	21	3	2	1
100	134,132	534	8	1,400,000	2	2, 21	3	2	1
101	13,900	162	6, 8	803,200	1	2	3	2	1
102	35,500	393	8	429,200	2	2, 21	1	1	1
103	32,100	93	2, 6	528,500	1	2, 21	3	2	1
104	73,100	629	1	1,900,000	2	2	3	2	1
105	370,900	3712	5, 8	7,700,000	2	3	3	2	1
106	223,700	922	5	3,100,000	2	3	3	2	1
107	190,800	1093	8	2,900,000	2	3	3	1	1
108	60,800	189	6	462,500	2	3	3	2	1
109	26,400	408	6	349,600	2	3	3	2	1
110	61,900	270	8	626,300	2	3	3	2	1
111	36,700	391	8	394,100	2	3	3	2	1
112	39,600	958	8	558,172	2	3	3	2	1
113	20,100	300	5	602,900	2	3	3	1	1
114	77,500	277	6	812,600	2	4	3	2	1
115	44,000	806	2, 6	490,500	2	4, 22	3	2	1
116	34,900	198	6	353,300	2	4	3	1	2
117	29,700	127	1	767,600	2	4	3	2	2
118	27,400	411	1	264,600	2	4	3	2	1
119	41,100	196	1	345,800	2	4	3	2	1
120	476,000	878	5	3,800,000	2	4	3	1	1
121	25,500	97	2, 6	170,300	1	6	3	2	1

**Table 3:** Number code for categories shown in Table 2. Occupational group, advertisement, ingredient, source of reference, misinformation, and language. Because of the formality for the category occupational group, the number 3 for nursing is mentioned, although it was not present in this sample

Occupational group	Advertisement	Ingredient	Source of reference	Misinformation	Language
1 = Doctor 2 = Pharmacist 3 = Nursing 4 = News channel 5 = Podcast 6 = Influencer 7 = Other expertise 8 = Unknown/no expertise 9 = Scientist	1 = Advertisement 2 = No advertisement	1 = Estrogen/Gestagen 2 = Levonorgestrel 3 = Testosterone/Derivatives 4 = Sildenafil 5 = Fentanyl 6 = Cannabis 7 = LSD 8 = MDMA 9 = Drugs unspecific 10 = Psilocybin 11 = Opioids 12 = Cocaine 13 = Methamphetamine 14 = Antidepressants 15 = Alcohol 16 = Benzodiazepines 17 = Ketamine 18 = Ibuprofen 19 = Heroin 20 = Naloxone 21 = Ulipristal acetate 22 = Glycerol trinitrate	1 = One source 2 = Several sources 3 = No source	1 = Misinformation 2 = No misinformation	1 = English 2 = German

## Results and discussion

### Advertisement

**Fig. 2:**
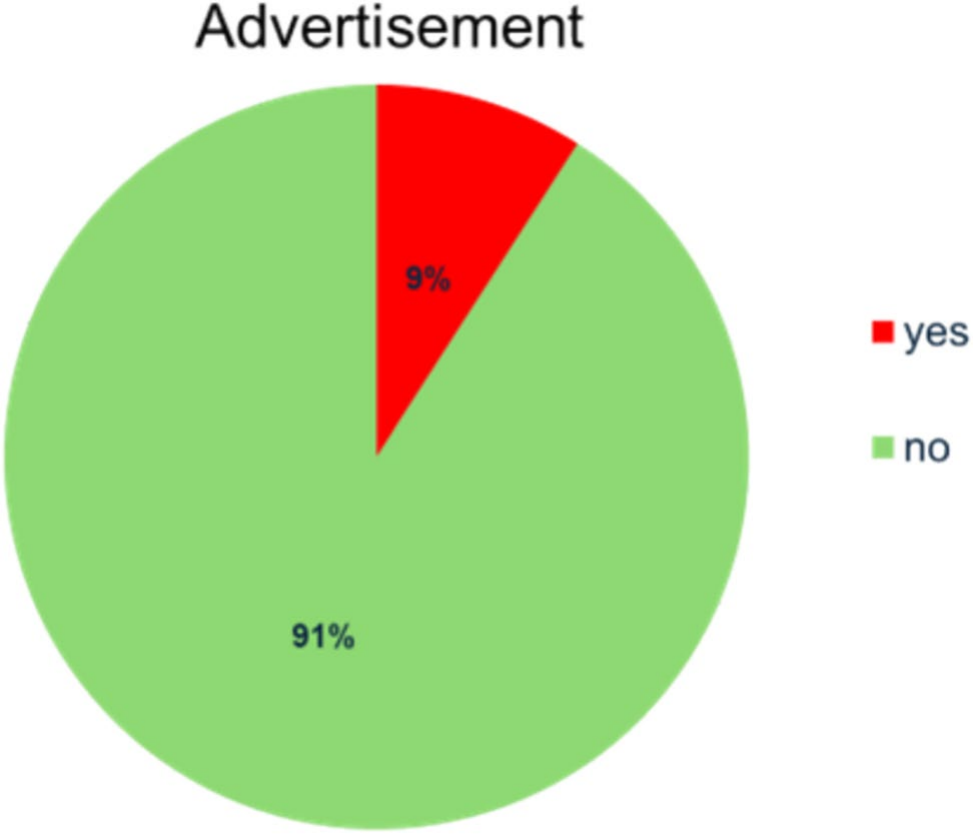
Advertisement as a pie chart

As shown in Fig. 2, 11 (9%) of the 121 samples contained a form of advertisement. Only advertisements concerning pharmacological content were considered. 110 (91%) posts did not include any advertisement. The relationship between occupational group and advertisement was investigated. Three (42.86%) posts created by pharmacists and 11 (20.76%) posts created by influencers included advertisements. One (4.76%) video created by the occupational group “unknown/no expertise” also contained advertisement. Because there was only one post created by a scientist that also included advertisement, the results of 1 (100%) are not representative. Videos produced by other occupational groups did not contain advertisement. A recent study has shown similar results about influencers advertising supplements on Instagram (Ricke and Seifert 2024). Sixty-three percent of posts analysed contained discount codes for said supplements advertised by influencers (Ricke and Seifert 2024).

**Fig. 3:**
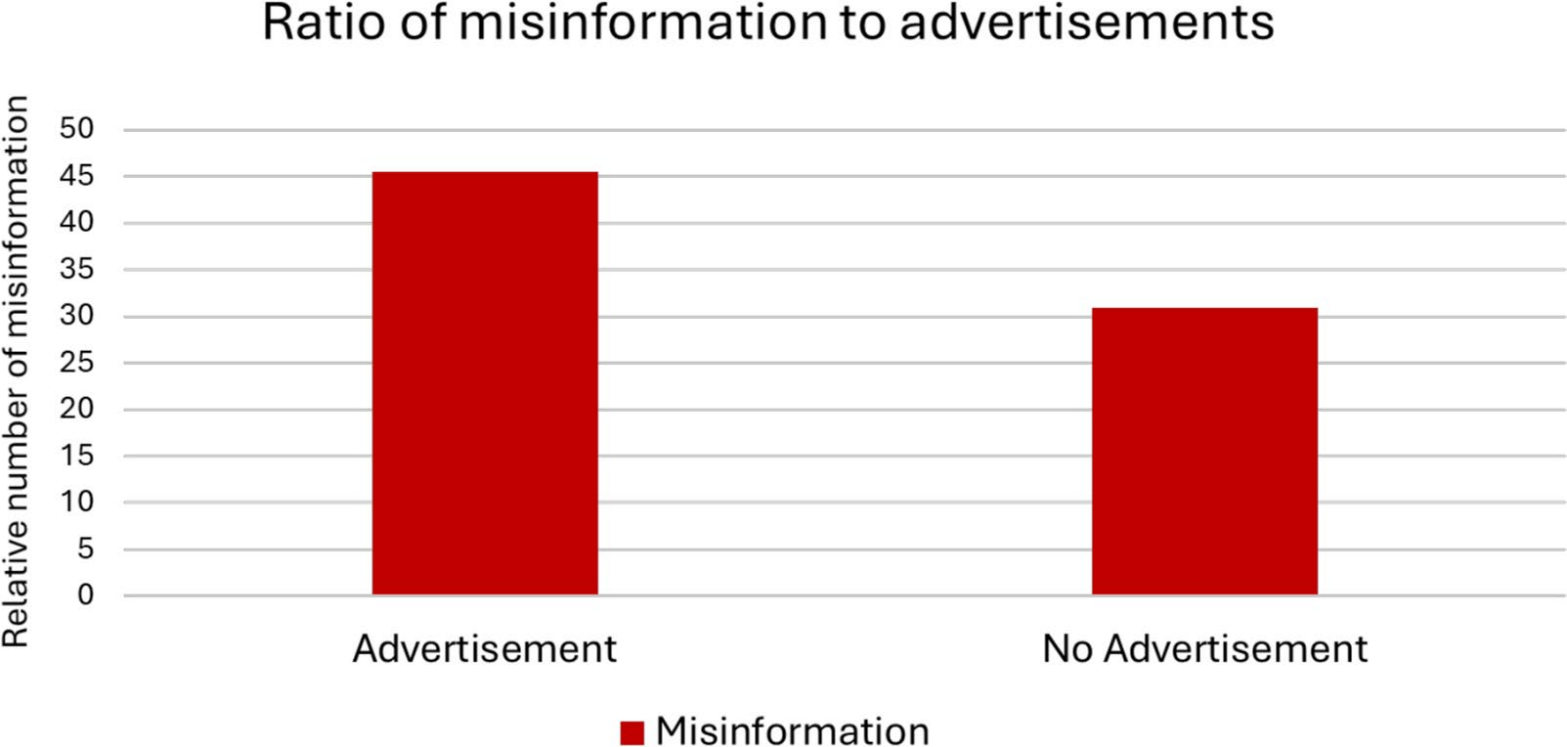
Relation between misinformation and advertisements as bar chartgiven as a percentage

Apart from the occupational group, we additionally explored the relationship between advertisements and misinformation present in the video. The results are shown in Fig. 3. The differences in misinformation among items with and without advertisement were not statistically significant in this study (chi^2^ test, *p* = 0.3250; Fisher’s exact test *p* = 0.3299). Even though results in this category were not significant, the phenomenon of advertisement and misinformation is shown widely across social media platforms and needs to be further investigated (Zenone et al. 2021).

### Source of reference

**Fig. 4:**
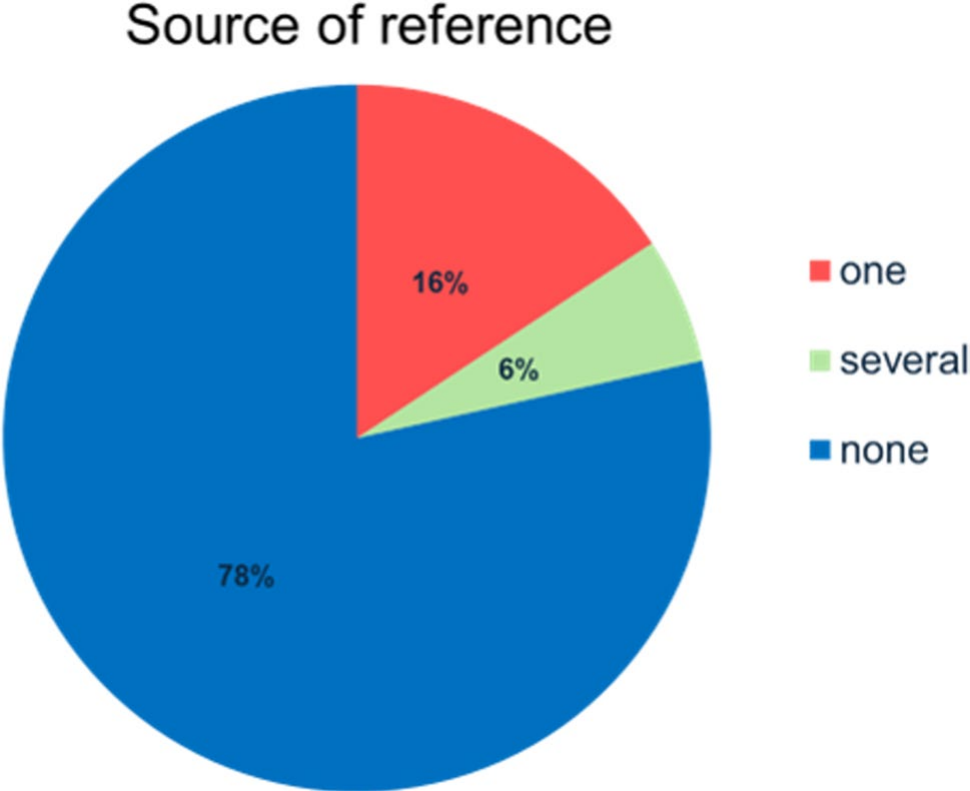
Source of reference as a pie chart

**Fig. 5:**
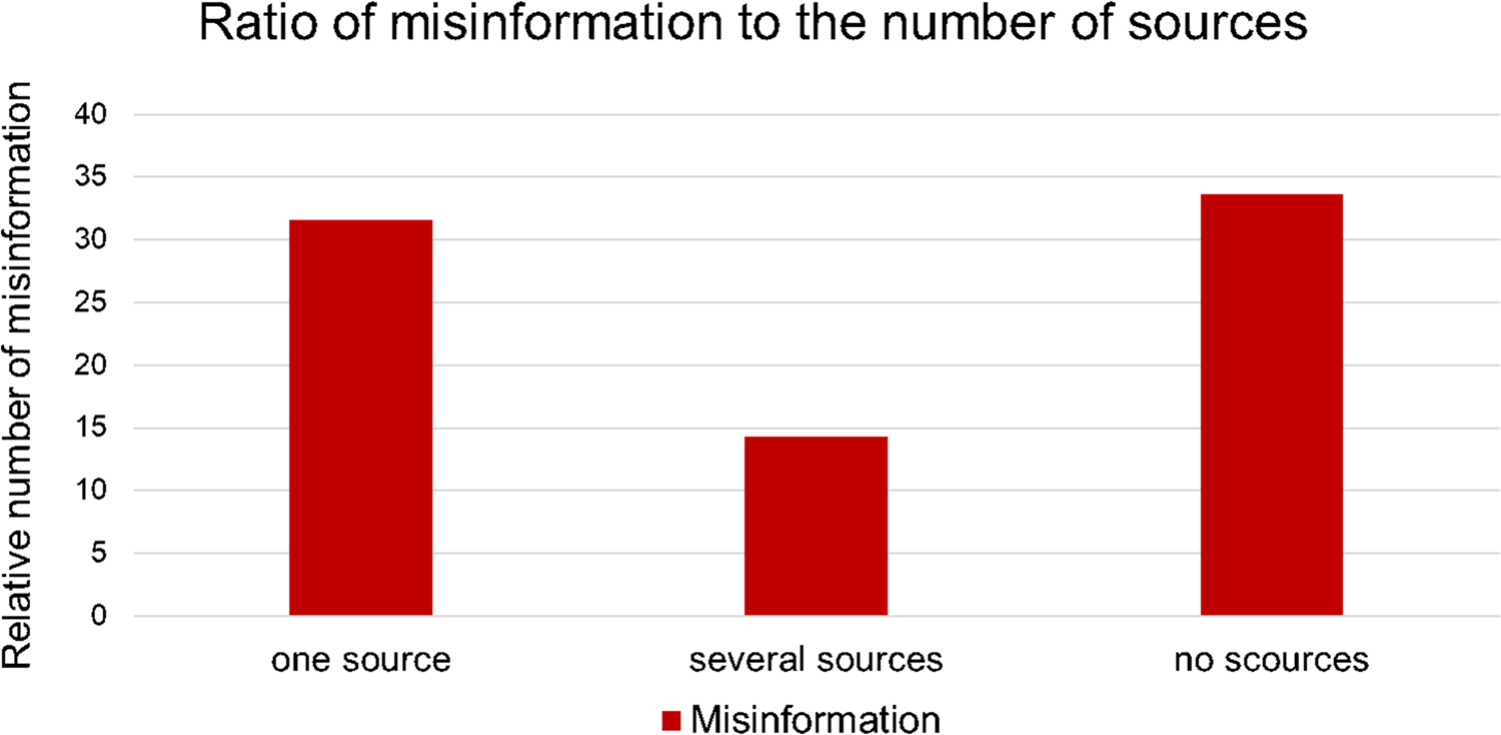
Relation of misinformation to sources as a bar chart given in percentage

19 (16%) of 121 posts included one source of reference. Only 7 (6%) contained more than one source of reference. The majority with 95 (78%) did not mention any source of reference (Fig. 4). The subcategory containing the highest number of posts with misinformation (33,68%) was no source of reference (Fig. 5). Videos with one source of reference contained 31.57% of misinformation among the posts. The subcategory with the least amount of misinformation (14.29%) was several sources of reference. The differences in misinformation were not statistically significant in this study (chi^2^ test, *p* = 0.5690; Fisher’s exact test, *p* = 0.66739. Thus, it is not possible to draw definitive conclusions regarding the influence of the source of reference on misinformation.

#### Occupational group

**Fig. 6:**
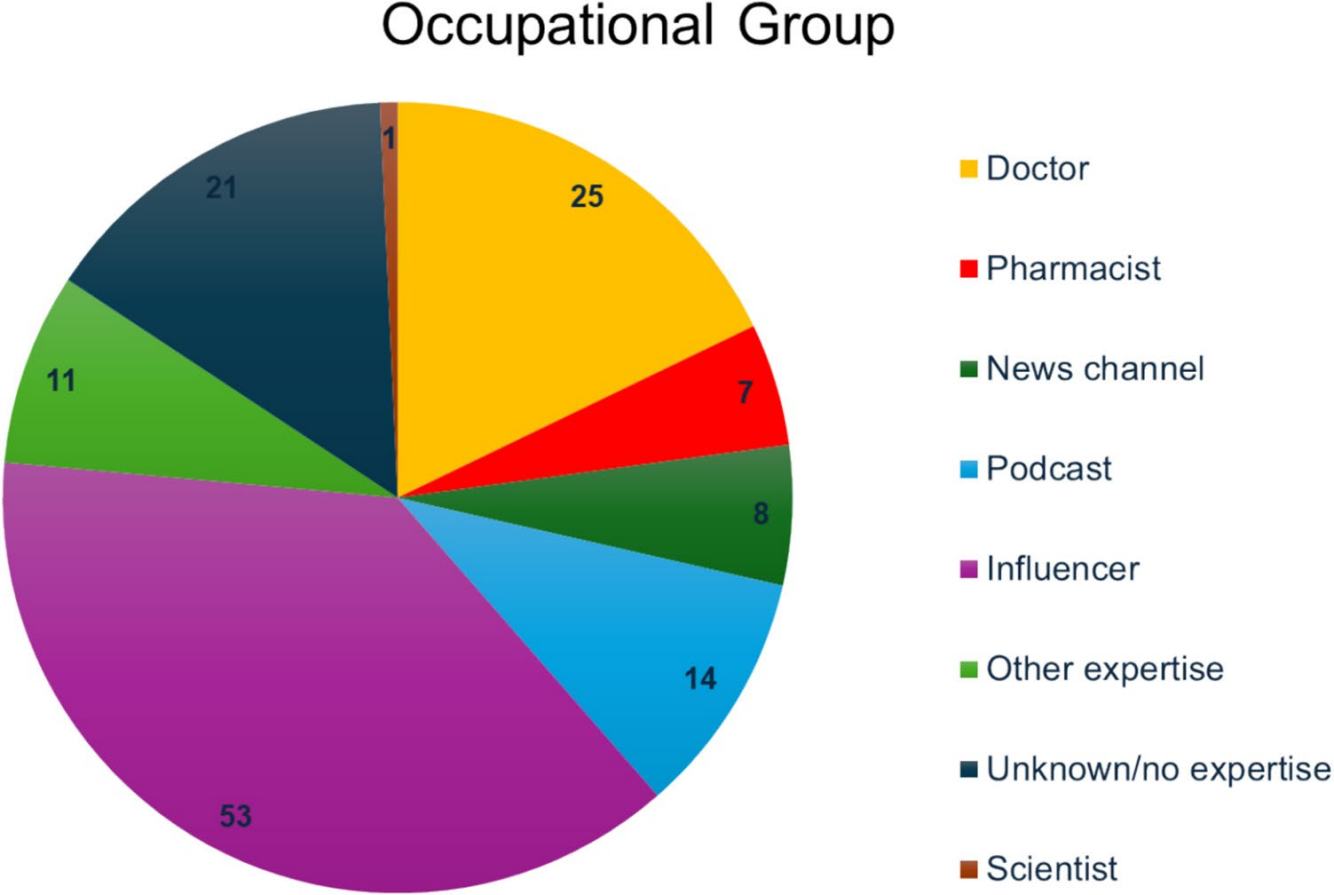
Occupational groups shown as a pie chart

Most posts were created by influencers (53) (Fig. 6). Doctors were the second largest group with 25 videos. Twenty-one creators were categorised as unknown/no expertise. Thus, most videos are created by people without any expertise or who have financial interests in creating content. This could result in lower quality of information and large spreading of misinformation among lays.

### Misinformation

Sixty-eight percent of 121 posts did not present any misinformation. Thirty-two percent of videos contained some form of medically related misinformation. English posts included misinformation in 33.3% of videos and German posts contained 25% misinformation. This difference was not statistically significant (chi^2^ test, *p* = 0.5064; Fisher’s exact test, *p* = 0.57959. Posts containing common terms (32.81%) were not significantly more likely to contain misinformation than posts using medical terms (31.58%). Of the videos that used moving visual animation technique, 43.48% contained misinformation. Posts that did not include animation only contained 29.59% misinformation. A recent study about dietary supplements found that two-thirds of posts analysed exceeded the nationally established recommendations for maximum dosage (Ricke and Seifert 2024). While supplement overdoses can also cause detrimental effects, false claims about medicinal products as analysed in this study could be more dangerous or even fatal. Examples of misinformation conveyed are shown in Table 4. Since one-third of our analysed sample contained false claims, it is essential to improve control mechanisms and safety regulations for TikTok users. Most of them are young people who are prone to be influenced by these videos which might be dangerous to their health and education. Influencers make promises of efficacy and trivialize side effects (Ricke and Seifert 2024). Young people are known to look for health-related information on social media more frequently over the last few years (Ricke and Seifert 2024). Our findings are congruent with the findings of other studies. One study investigated 166 TikTok videos in terms of Covid-19-related misinformation (Baghdadi et al. 2023). The study showed that 22% contained moderate level of misinformation and 11% contained high-level misinformation (Baghdadi et al. 2023). Additionally, posts containing high-level misinformation seemed to achieve more engagement among viewers (Baghdadi et al. 2023).

**Table 4:** Misinformation in content presented on TikTok

File number	Misinformation	Occupational Group	Correct information	Source	Misinformation category	Language
2	MDMA, LSD, and psilocybin have the same pharmacological effects	Podcast	MDMA, LSD, and psilocybin are different drugs and do not have the same effects	1. Nutt et al. (2010) 2. Hase et al. (2022)	Incorrect information	English
5	Benzodiazepines and alcohol cause one of the most dangerous drug interactions	Doctor	Sedating effects on the CNS may be potentiated but there are many other very dangerous drug/drug interactions that can be fatal	1. Roque Bravo et al. (2022) 2. Freissmuth et al. (2020) 3. Gudin et al. (2013)	Incorrect information	English
9	CBD oil helps with anxiety. Pets get intoxicated by licking Voltaren® off of their owner’s skin. Ibuprofen causes inflammation	Podcast	CBD oil effect on anxiety is not proven. There are no studies concerning pet intoxication with Voltaren®. There is only one paper discussing the possibility of Ibuprofen worsening arthritis inflammation. Ibuprofen in general is known to have anti-inflammatory effects	1. Hazekamp (2018) 2. Spinella et al. (2021) 3. Legare et al. (2022) 4. Soleymanpour et al. (2021) 5. Dunayer (2004) 6. Nissen et al. (2016) 7. Luitjens (2022) 8. Rogers and Aronoff (2016)	Lack of evidence	English
10, 11, 14	CBD oil helps with anxiety	Influencer	CBD oil effect on anxiety is not proven	1. Hazekamp (2018) 2. Spinella et al. (2021) 3. Legare et al. (2022) 4. Soleymanpour et al. (2021)	Lack of evidence	English
18	Cannabis use does not influence lung cancer risk	Influencer, scientist	Studies have shown that there is a possible relation between lung cancer and cannabis use. The effects still need to be investigated	1. Jeffers et al. (2024) 2. Connor et al. (2021) 3. Hoch et al. (2019) 4. Baumeister et al. (2021) 5. Tashkin (2014)	Incorrect information	English
21	CBD oil does not cause positive drug test results. There are no side effects. It helps with anxiety and muscle pain	Influencer	The mentioned products contain various levels of THC doses. Studies have shown that through THC contamination, CBD oil use can lead to positive drug test results. CBD can have side effects and its effect on anxiety is not proven. There are no studies showing that it helps with muscle pain	1. Arkell et al. (2020) 2. Johnson et al. (2022) 3. Legare et al. (2022) 4. Spinella et al. (2021)	Lack of evidence	English
23	Cannabis eases Parkinson’s symptoms like tremor	Unknown/no expertise	There are no studies proving this claim. Case numbers are very little so no assumption can be made	1. Legare et al. (2022) 2. Hoch et al. (2019) 3. Figura et al. (2022) 4. Singh et al. (2023)	Lack of evidence	English
26	Pictures show brain atrophy in cannabis users. Cannabis limits blood flow to the hippocampus and impairs memory	Influencer	There is no study showing pictures confirming this claim. The website mentioned is not scientifically verified. Studies show influence on hippocampus and memory but only in terms of acute consumption	1. Filbey et al. (2018) 2. Kloft et al. (2020) 3. Kutlu and Gould (2016)	Incorrect information	English
27	Cannabis increases risk for psychosis four times	Doctor, podcast	There are studies confirming a higher risk for psychosis but we could not find a study describing the mentioned numbers	1. Bara et al. (2021) 2. Connor et al. (2021) 3. Hoch et al. (2019) 4. Hill et al. (2017) 5. Hamilton (2017) 6. Bossong and Niesink (2010)	Incorrect information	English
35	MDMA does not harm the brain and has only been made illegal because of its concurrence to the alcohol industry	Unknown/no expertise	MDMA use does harm the brain. There is no evidence that the illegalisation of MDMA was related to the alcohol industry	https://www.nytimes.com/1985/06/01/us/us-will-ban-ecstasy-a-hallucinogenic-drug.html 1. Kalant (2001) 2. Mustafa and Mohamad (2019) 3. Pentney (s2001)	Incorrect information, lack of evidence	English
37	MDMA reduces dopamine. When used therapeutically, it does not cause “hangover symptoms”	News channel	Recent studies show that MDMA increases dopamine. Studies investigating treating PTSD with MDMA describe hangover	1. Feduccia and Mithoefer (2018) 2. Freissmuth et al. (2020) 3. Kalant (2001) 4. Mitchell et al. (2023) 5. Parrott (2002)	Incorrect information	English
39	A study showed impaired memory in MDMA users. They forgot 25% more items on the list than non-users	Unknown/no expertise	There is no study confirming this	1. Freissmuth et al. (2020) 2. Kalant (2001) 3. Mustafa and Mohamad (2019) 4. Pantoni et al. (2022) 5. Seifert (2018)	Incorrect information	German
40	175 people die every day from fentanyl poisoning. Two people went into cardiac arrest because of performing CPR on fentanyl users and therefore inhaling the drug	Influencer	There is no official source confirming these numbers. Also, there is no official source describing this phenomenon of overdosing while doing CPR. Toxicologists have shown that it is not possible to inhale this much Fentanyl to reach an overdose this quickly	1. Ciccarone (2021) 2. Del Pozo et al. (2022) 3. Nelson and Schwaner (2009) 4. Patocka et al. (2024) 5. Rauf et al. (2021) 6. Volkow and Blanco (2021)	Incorrect information	English
43	There is no safe dose of fentanyl. It is impossible to know if a drug is laced with fentanyl	Influencer	In a therapeutic setting like anaesthesia, there is a safe dose of fentanyl. There are test kits to test drugs on fentanyl content	1. Freissmuth et al. (2020) 2. Norman et al. (2023)	Incorrect information	English
45	The video includes a diagram showing US deaths by drug overdose	Other expertise	The shown numbers could not be verified; the NIDA shows different numbers	https://nida.nih.gov/research-topics/trends-statistics/overdose-death-rates	Incorrect information	English
51	Number of national fentanyl drug overdose deaths in the USA	Influencer	The shown numbers could not be verified; the CDC shows different numbers	CDC (2021)	Incorrect information	English
52	The deathliest and most addicting drug is sugar. The second are tobacco and alcohol. Fentanyl is not even close	Doctor	Fentanyl is the number one cause of death for people aged 18 to 45 in the USA. Also, drugs like Heroin have much higher addiction tendencies and mortality	1. Nutt et al. (2010) 2. Patocka et al. (2024)	Incorrect information	English
62	Size and weight affect the duration of LSD effects	Pharmacist	There are no studies showing that size has any influence at all. Weight also seems to have little to no effect	1. Dolder et al. (2017) 2. Freissmuth et al. (2020) 3. Holze et al. (2021) 4. Jastrzębski et al. (2022) 5. Kuc et al. (2022) 6. Seifert (2018)	Lack of evidence	English
67	LSD does not cause addiction	Influencer	LSD does not cause physical addiction but can cause psychological addiction	1. Freissmuth et al. (2020) 2. Holze et al. (2021) 3. Klock et al. (1975) 4. Nichols (2016) 5. Schmid and Liechti (2018)	Incorrect information, generalization	English
68	Video shows an illustration of a birth control pill stopping the sperm from entering the egg	Influencer	This is not correct, hormonal birth control prevents ovulation	Freissmuth et al. (2020)	Incorrect information	English
75	Hormonal birth control causes you to gain less muscle mass. Taking hormonal birth long-term cycle is unhealthy. It can cause galactorrhoea. Progesterone implants can cause PCOS	Influencer	Hormonal birth control influencing muscle gain is not proven. There are medicinal advantages to a long-term cycle use of contraceptives. There is no evidence supporting that there is a correlation between contraceptives and galactorrhoea. Birth control does not cause PCOS and is even a possible therapeutic option	1. Atluri et al. (2018) 2. Freissmuth et al. (2020) 3. Lopez et al. (2016) 4. Nolan et al. (2024) 5. Sheehan (2004)	Lack of evidence, incorrect information	English
79	Hormonal birth control causes Estrogen dominance. It influences the vaginal flora and the gut microbiome. It increases risk for ovarian cancer. Lactose consumption can trigger Hashimoto’s thyroiditis	Unknown/no expertise	There are no studies describing an Estrogen dominance. The findings for changes in the vaginal flora are incongruent and not proven. There is no association to the gut microbiome. Hormonal birth control has protective effects concerning ovarian cancer. There are no studies suggesting that Hashimoto thyroiditis is triggered by Lactose; there is a possibility that the absorption of L-thyroxine can be decreased	1. Aminzadeh et al. (2016) 2. Ampatzis (2022) 3. Coelingh Bennink et al. (2017) 4. Freissmuth et al. (2020) 5. Kamani et al. (2022) 6. Krog et al. (2022) 7. Lee and Syed (2022) 8. Seitz (2023) 9. Sung et al. (2022) 10. Usai-Satta et al. (2022) 11. Westhoff et al. (2013)	Lack of evidence, incorrect information	English
81	Women should not be on hormonal birth control. Your periods are not supposed to just stop. The copper IUD causes the whole body to be inflamed all the time	Podcast, influencer	There are advantages for hormonal birth control and long-term cycle use, for example endometriosis. The copper IUD only has local effects and does not cause inflammation in the whole body	4. Seitz (2023)	Incorrect information, generalization	English
83	Non-hormonal birth control for men is going to have no side effects. Several side effects like trouble breathing and chest pain	Influencer, unknown/no expertise	Non-hormonal contraception can also have side effects. Many of the mentioned side effects are exaggerated for entertainment purposes	1. Balbach et al. (2023) 2. Freissmuth et al. (2020) 3 Seitz (2023)	Generalization, incorrect information	English
84	Women on birth control make different mate choices and find different body scents appealing than they normally would. The scent selection takes place because of differences in immune systems. Women on birth control often choose actively against their natural instincts. That is why divorce rates are so high nowadays	Podcast, influencer	The mentioned study took place in 1995. Recent studies contradict these findings. There is no proven effect on choice based on scent and immune system. Any connection to divorce rates is not proven	1. Cobey et al. (2016) 2. Havlíček et al. (2020) 3. Wedekind (1995)	Lack of evidence	English
85	Hormonal birth control can cause prolactinoma. Symptoms are hot flushes	Influencer	There is no evidence connecting birth control to prolactinomas. Hot flushes are also not described in literature	1. https://www.niddk.nih.gov/health-information/endocrine-diseases/prolactinoma 2. Vroonen et al. (2019) 3. Wildemberg et al. (2021)	Lack of evidence	English
86	The leading death in women is heart disease. Birth control increases the risk and it is possible that heart disease numbers increase because of birth control	Podcast, influencer	Heart disease is also known to increase in the male population. There are no studies showing a correlation to birth control but rather a change in lifestyle	1. Naghavi et al. (2024) 2. Seitz (2023)	Lack of evidence	English
89	Hormonal birth control makes the patient’s life worse. Women who smoke should never be given hormonal birth control	Doctor, podcast, influencer	This is an incorrect generalization. Smoking in general is not an absolute contraindication but a relative one. It depends on other health factors and cigarettes per day	Seitz (2023)	Generalization	English
92	Side effects of hormonal birth control include depression which women often realize after they stop taking it because they got used to the side effects	Influencer	Hormonal birth control can cause depression but there is no evidence that side effects become apparent after discontinuation	1. Gemzell‐Danielsson et al. (2022) 2. Mengelkoch et al. (2024) 3. Segarra et al. (2023)	Lack of evidence	German
94	Hormonal birth control is a modern poisoning of women. Envitox USA classified it as totally cancerous. Synthetic estrogens are one of the groups with the highest destruction potency. 25–60% of users show differences in brain structure. 100% of users show 6% reduced hypothalamus volume. One-third of users have a disease of the thyroid	Unknown/no expertise	Envitox did not classify hormonal birth control as cancerous. It also does not poison women. Some studies show influence on brain structure but without any of the claimed numbers. There is an association with thyroid diseases, but again, there are no studies describing these numbers	1. Qiu et al. (2021) 2. Seitz (2023) 3. Song et al. (2023)	Lack of evidence, incorrect information, generalization	German
95	Hormonal birth control causes PCOS and prolactinoma	Influencer	Hormonal birth control does not cause prolactinoma or PCOS	1. Sheehan (2004) 2. Siddiqui et al. (2022) 3. Vroonen et al. (2019) 4. Wildemberg et al. (2021)	Incorrect information	English
97	No women should ever take emergency contraception. It increases risk for ectopic pregnancies. The rise of ectopic pregnancies in Nairobi is due to emergency contraception	Doctor	This generalization is medically incorrect. Emergency contraception increases ectopic pregnancy risk only when while usage of it a pregnancy develops. Emergency contraception itself does not increase the risk. There are no studies showing a connection between Nairobi ectopic pregnancies and emergency contraception	1. Mutiso and Mugambi (2023) 2. Restaino et al. (2022) 3. Seitz (2023) 4. Shulman (2010) 5. Zhang et al. (2015) 6. Zhang et al. (2023)	Lack of evidence, generalization	English
102	Emergency contraception increases breast cancer risks by 44%	Unknown/no expertise	There are no studies supporting this claim	Fitzpatrick et al. (2023)	Lack of evidence, incorrect information	English
107	When a person exercises intensively, testosterone levels drop a lot sometimes to the level of a castrated man	Unknown/no expertise	Testosterone can decrease while exercising but not to the level of a castrated man	1. Anderson et al. 2016) 2. Bond et al. (2022) 3. Zouhal et al. (2022)	Incorrect information	English
113	Everything gets better with testosterone. Testosterone levels have dropped 50% in the last 20 years	Podcast	Testosterone therapy also has side effects. There is no study supporting these numbers	1. Bond et al. (2022) 2. Lokeshwar et al. (2021) 3. Snyder et al. (2018)	Incorrect information, generalization	English
116	Side effects of Viagra® are fatal hypotension. It can cause Priapism which can cause one to lose the penis	Influencer	Fatal hypotension is mostly described in combination with glycerol trinitrate. Erection is still stimulus-dependent, and therefore, in normal dosages, sildenafil does not cause priapism	1. Freissmuth et al. (2020) 2. Pușcașu et al. (2023) 3. Seifert (2018)	Incorrect information	German
120	10 mg of Viagra® resulted in priapism and effects as late as 5 h later. It caused numbness in the arms	Podcast	10 mg is below the standard dose. The first effects are seen after 30 min and last up to four hours. It does not cause priapism. Numbness is also not a known side effect	1. Fink et al. (2002) 2. Freissmuth et al. (2020) 3. Rasmussen et al. (2007)	Incorrect information	English

As shown in Table 4, many claims were made against scientific evidence. This is an immediate threat to the user’s health. For example, one influencer linked her newly diagnosed prolactinoma to her previous progesterone implant and advised users to do their own research and not trust doctors’ advice. This is misleading since there is no scientific evidence describing progesterone implants as a possible cause for prolactinomas (Wildemberg et al. 2021). This particular video got 10.7 million views on TikTok.

Influencers gain the trust of their often young followers and make promises linked to certain achievements for products advertised (Ricke and Seifert 2024). A relationship characterised by dependence is the result (Ricke and Seifert 2024). Children liking an influencer make it more likely that they will believe the information presented and imitate behaviour (Coates et al. 2019). Therefore, posts like the one mentioned are even more dangerous for this group, especially when they get this much attention and interaction. People could be afraid of using birth control because they fear developing prolactinoma which may lead to more unwanted pregnancies.

CBD is also a frequently discussed ingredient on social media. The market for these products has increased rapidly (Soleymanpour et al. 2021). Oils and tinctures are exceptionally popular (Soleymanpour et al. 2021). The four main therapeutic claims made are effects on anxiety, stress, pain, and sleep disorders (Soleymanpour et al. 2021). But CBD has no proven effect on these symptoms (Spinella et al. 2021). There is no objective evidence supporting claims on social media, yet many posts of our sample promised immediate anxiety relief. They also advised users to take more than the recommended dosage if they do not feel the desired effects.

Some posts also suggested that CBD oil has no side effects at all. Studies investigating CBD use are still very limited (Soleymanpour et al. 2021). Therefore, consequences of prolonged use are still unknown (Hazekamp 2018). Some studies describe side effects such as diarrhoea and direness (Iffland and Grotenhermen 2017). Aside from this, recent research has shown that especially unregulated CBD products are labelled as “THC-free” but are contaminated with levels of THC up to 0.656 mg/mL (Johnson et al. 2022). This unintentional consumption of THC can also cause various side effects or even addiction (Johnson et al. 2022).

Another example for dangerous misinformation occurred in a post discussing LSD. The creator made the generalised statement that LSD does not cause addiction. While it is correct that LSD does not seem to cause physical addiction, it has severe psychologically stimulating effects which may cause psychological addiction to the drug (Passie et al. 2008). Because participants in studies had already developed tolerance to the effects after a few doses, it can be assumed that someone dependent on these effects from a psychiatric perspective would need more LSD to reach the desired outcome (Passie et al. 2008). This shows that not only false claims but also generalisations can have dangerous consequences. Because of this post, users may view LSD as a “safe drug” and not fear adverse effects.

### Active ingredients/drugs

**Fig. 7:**
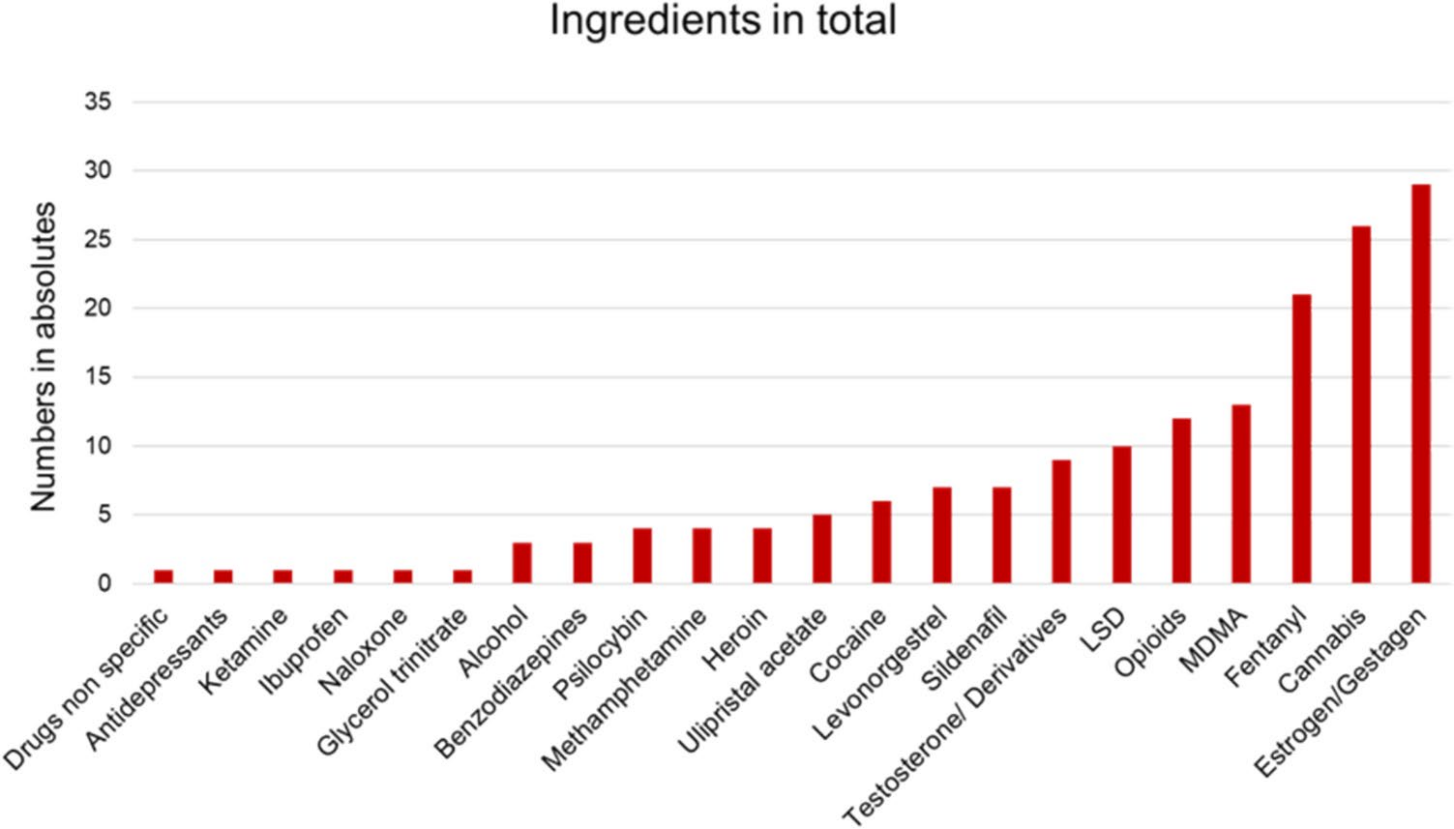
Active ingredients/drugs as a bar chart

The active ingredients mentioned were as follows: drugs nonspecific, antidepressants, ketamine, ibuprofen, naloxone, glycerol trinitrate, alcohol, benzodiazepines, psilocybin, methamphetamine, heroin, ulipristal acetate, cocaine, levonorgestrel, sildenafil, testosterone/derivatives, LSD, opioids, MDMA, fentanyl, cannabis, estrogen/gestagen. The ingredient discussed most frequently was estrogen/gestagen with 29 mentions (Fig. 7). Second was cannabis (26) and third was fentanyl (21). The ingredient class mentioned most often was illicit drugs with 66 mentions. Second were sex hormones (45) and third MOR-agonists (12).

### Source of reference and occupational group

**Fig. 8:**
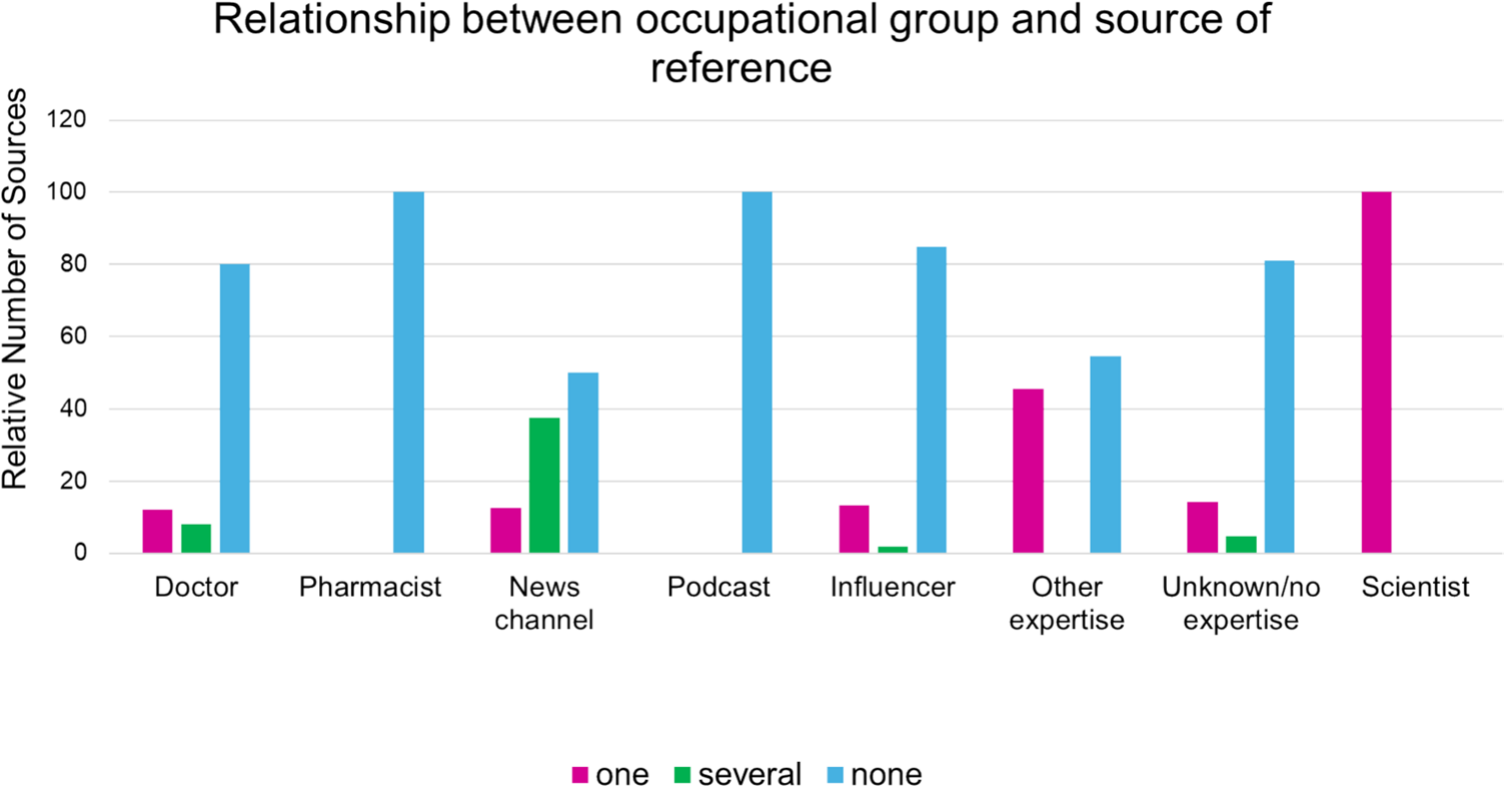
Relation of occupational group and source of reference as a bar chart

The two occupational groups “pharmacist” and “podcast” did not use any source of reference in this research. The group most likely to use several sources was “news channel” (37.5%). Since only one scientist was part of this study, the results concerning scientists are not reliable. The group that used one source of reference most often was the group “other expertise”. With 80% using no source of reference, “doctor” yielded similar results like “influencers” and people with “unknown/no expertise”. All results are shown in Fig. 8. This finding was statistically significant with a chi^2^ test *p* value of 0.003.

### Misinformation and occupational group

**Fig. 9:**
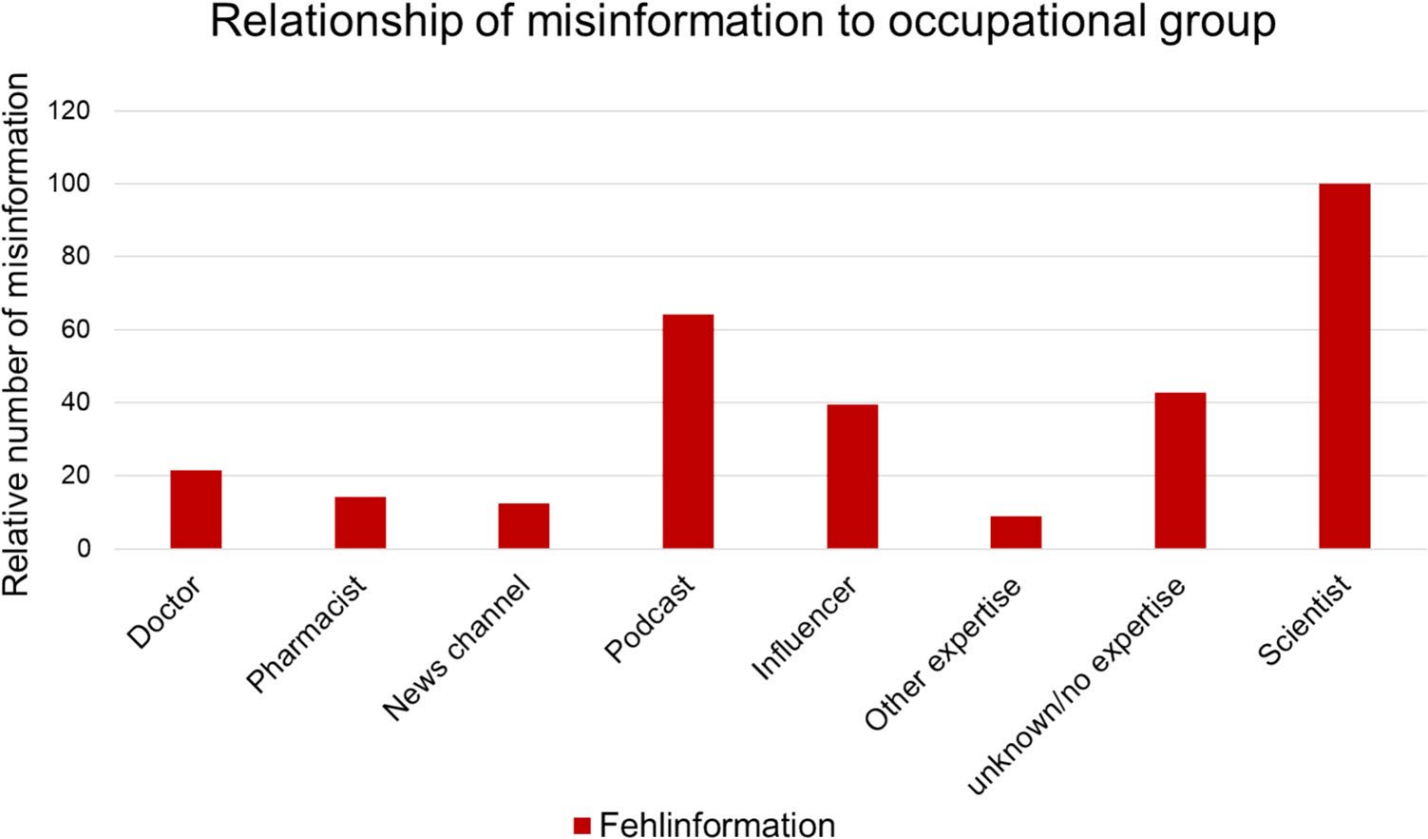
Relation of misinformation and occupational group as a bar chart

The relationship between presence of misinformation and occupational group was investigated. Every occupational group contained posts with misinformation. The occupational group containing the highest number of videos with misinformation was “influencers” with 21 posts (Fig. 9). “Podcasts” contained 9 and “unknown/no expertise” contained 8 videos including misinformation. This finding was statistically significant in our analysis with a chi^2^ test *p* value of 0.0191 and Fisher’s exact test p value of 0.0180. This shows that misinformation is more common in occupational groups without expertise. Qualifications and conflicts of interest are mostly unknown, so it is difficult for a user to estimate the quality of information (Zenone et al. 2021).

Videos created by officials or people with credible expertise are more likely to be viewed than others (Baghdadi et al. 2023). Hashtag searches for “medicine” and “doctor” were viewed 1.4 billion and 6.7 billion times (Comp et al. 2021). Thus, lay-friendly communication of official sources on TikTok can be an important way to oppose misinformation (Baghdadi et al. 2023). Official sources could also convey important information in cooperation via already existing popular accounts on TikTok (Comp et al. 2021). The short duration and compressed content on TikTok can achieve active learning within users (Comp et al. 2021). The use of hashtags results in a thread of posts about the same topic, which presents an opportunity for repetition learning (Comp et al. 2021).

### Misinformation and active ingredients/drugs

**Fig. 10:**
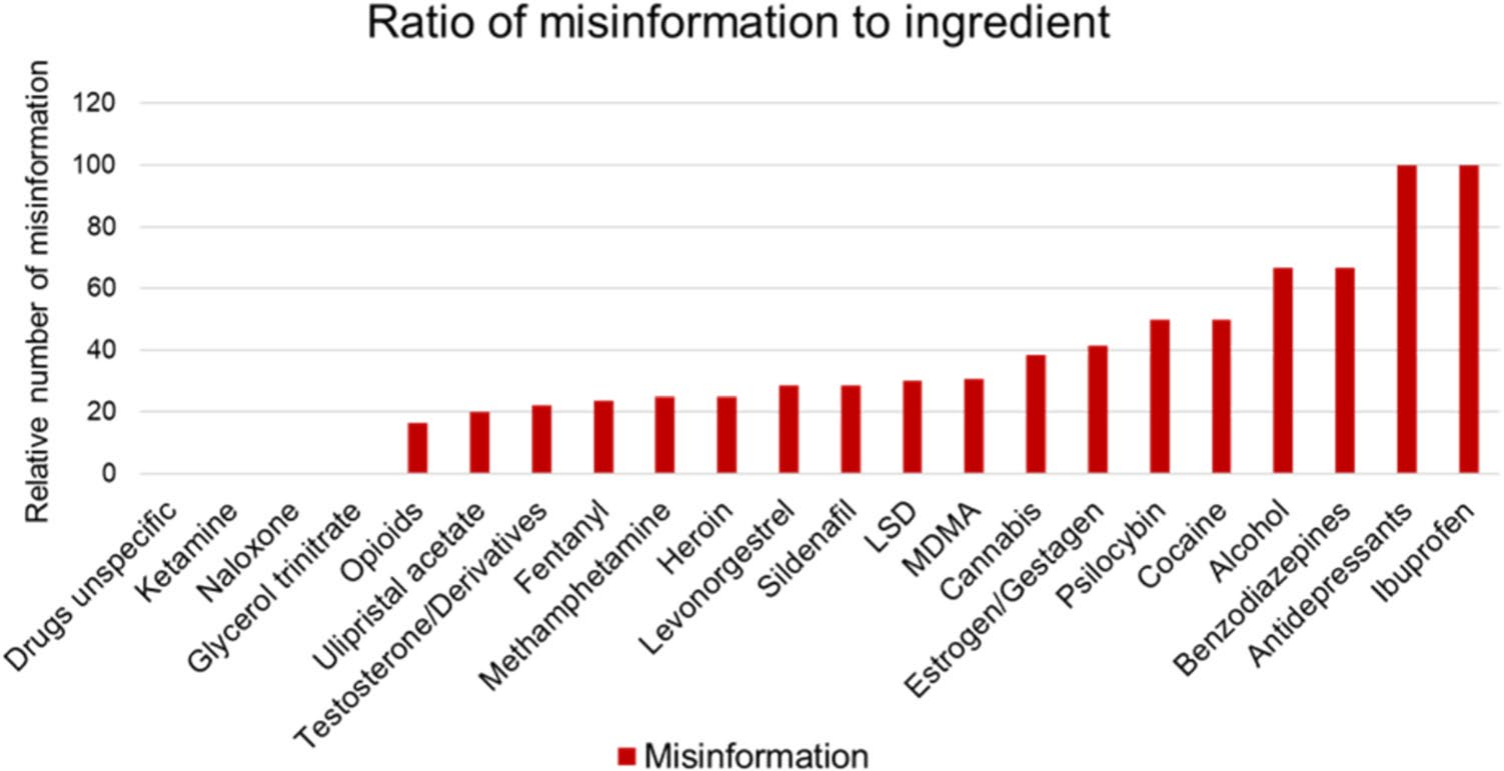
Relation of misinformation and active ingredient/drug as a bar chart

Since some ingredients like ibuprofen and antidepressants were only mentioned once, we focused on ingredients that were mentioned more than five times. The lowest relative number of misinformation was found in posts containing content about opioids (16.67%) (Fig. 10). Videos on ulipristal acetate (20%) and testosterone/derivatives (22.23%) contained slightly more misinformation. Fentanyl- (23.81%), levonorgestrel- (28.57%), and sildenafil- (28.57%) associated posts were more common to display of incorrect information. The occurrence of misinformation in posts about LSD (30%) and MDMA (30.77%) was even higher. Videos concerning cannabis (38.46%) were the third most common in this category. Second was estrogen/gestagen with a relative amount of misinformation of 41.38%. The relative number of misinformation was highest in videos concerning cocaine (50%). These findings were not statistically significant in this research (chi^2^ test, *p* = 0.8136). More studies are needed to confirm or negate these results. Table 5 provides a comprehensive summary of all statistical comparisons performed in this study.

**Table 5.** Results of the statistical analysis regarding the association between misinformation and other analysed factors. The table shows calculated *p* values. Statistically significant *p *values (*p* < 0.05) are highlighted

Parameters	Chi^2^ test—*p* value	Fisher’s exact test—*p* value
Advertisement and misinformation	0.3250	0.3299
Source and misinformation	0.5690	0.6673
Occupational group and misinformation	**0.0191**	**0.0180**
Ingredient and misinformation	0.8136	Sample too large

### Number of views and active ingredients/drugs, animation, and language

**Fig. 11:**
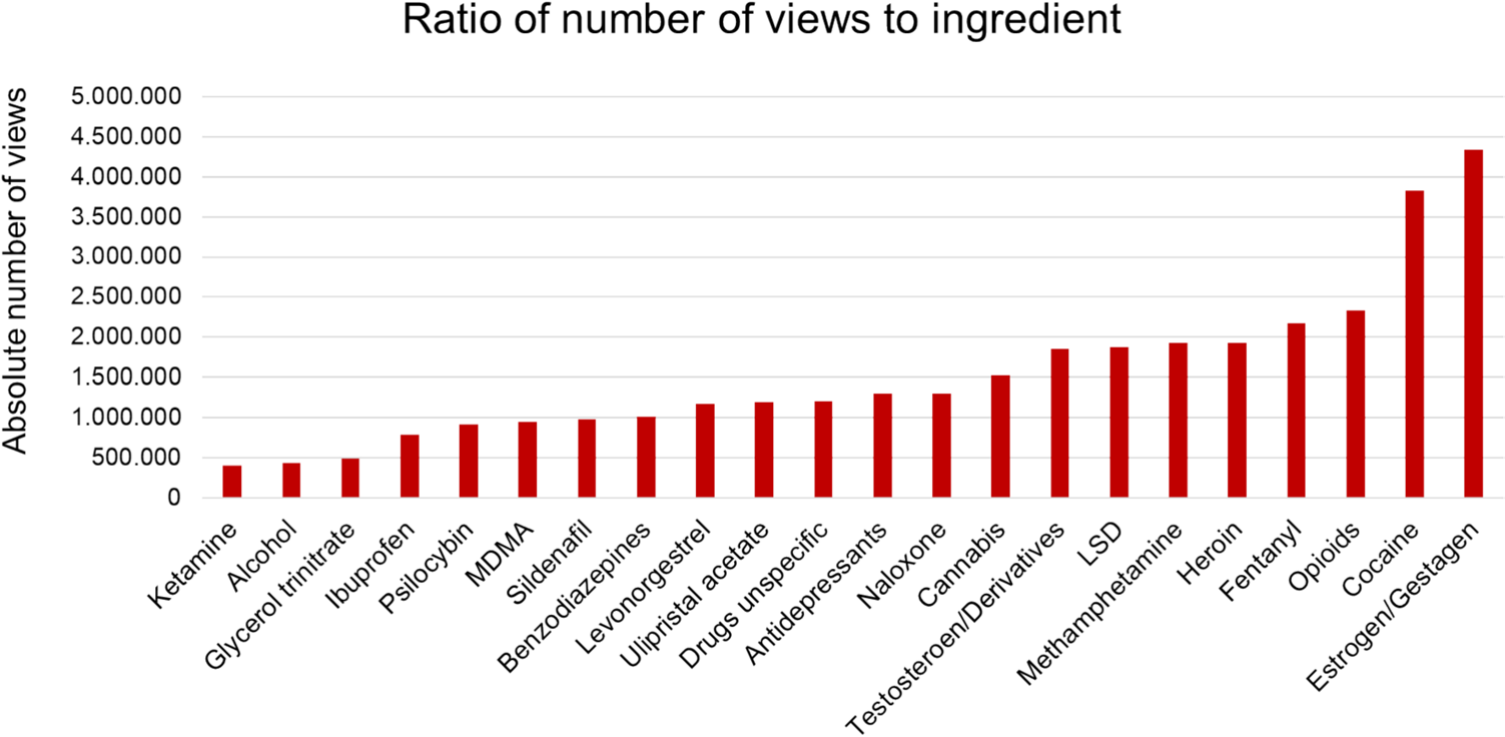
Relation of number of views and active ingredient/drug as a bar chart

To establish an understanding of which ingredients/drugs are most popular on TikTok, we analysed the number of views for each ingredient (Fig. 11). The most popular ingredient was estrogen/gestagen with 4.339.307 views. Second was cocaine (3.823.300) and third opioids with 2.335.256 views. This is concerning because misinformation spreads easily on TikTok and can dominate search results on a specific topic (Baghdadi et al. 2023). Since TikTok is mostly used by young people of lower income, it is possible that misinformation on highly popular topics like birth control influence health-related behaviours (Baghdadi et al. 2023). Although posts with high-level misinformation present are less likely to be viewed, they are still watched by millions (Baghdadi et al. 2023). A study found that one in four posts containing misinformation were commented to express the intention to change behaviour (Baghdadi et al. 2023).

**Fig. 12:**
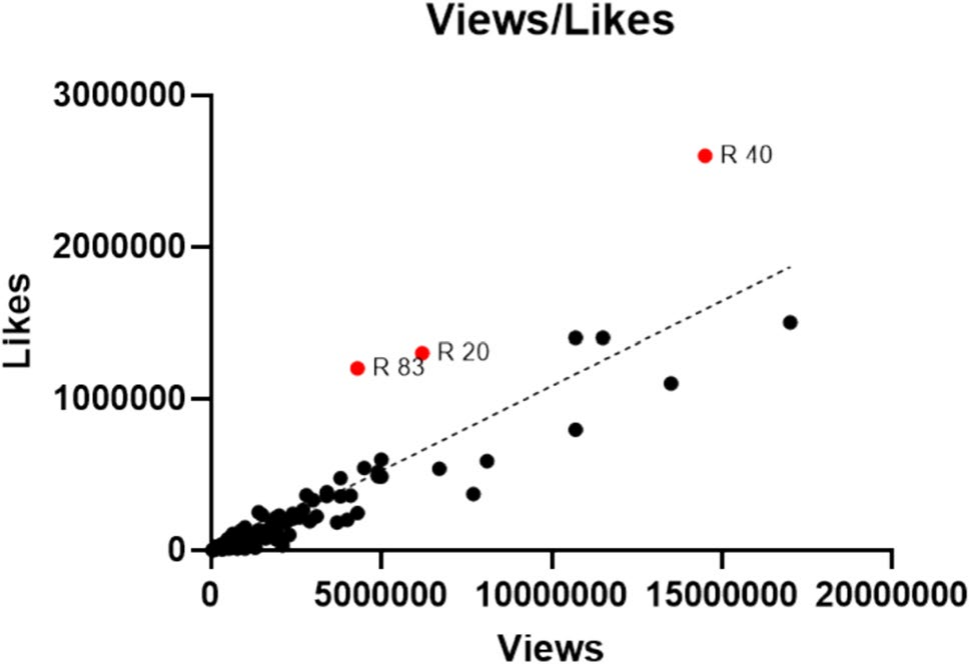
Linear regression concerning the relationship between views and likes as YX-diagram. The red dots represent highly liked posts and are labeled by their file number

**Fig. 13:**
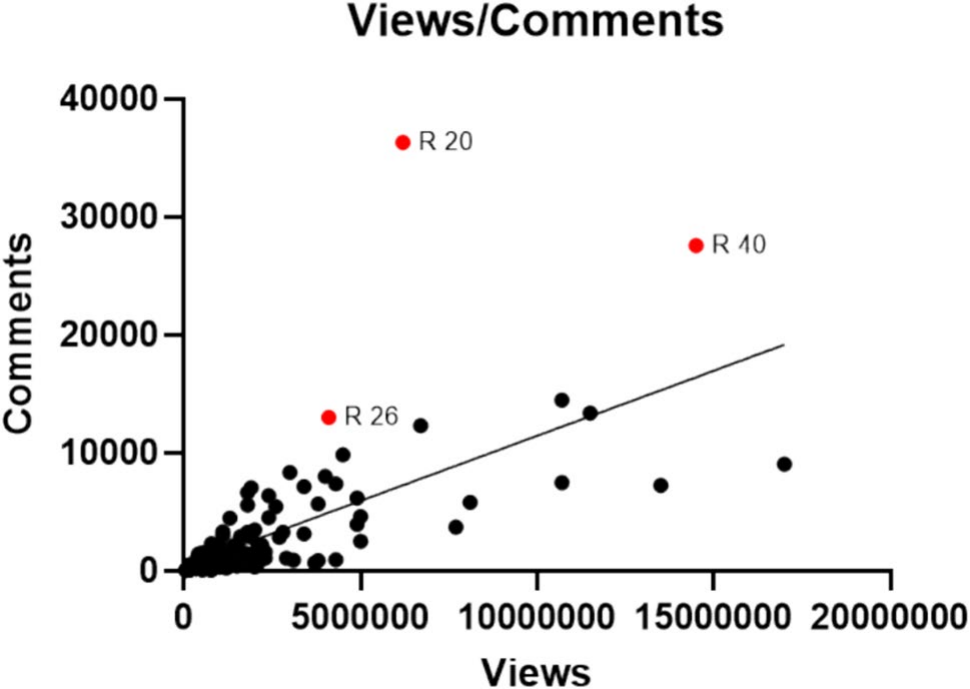
Linear regression concerning the relationship between views and comments as XY-diagram. The red dots represent highly commented posts and are labeled by their file number

We found a very strong positive correlation between the number of views and the number of likes (*r* = 0.8984, *p* < 0.0001). A linear regression showed that 80.7% of the variance in the number of likes could be explained by the number of views (*R*^2^ = 0.8070). There was a moderately strong correlation between the number of views and the number of comments (*r* = 0.6732, *p* < 0.0001), with *R*^2^ = 0.4532 indicating a lower explained variance. Results are shown in Figs. 12 and 13.

Our results suggest a different user behaviour considering likes and comments. This phenomenon has been investigated in previous studies as well. A user in a study in 2025 stated that even when a user likes controversial posts, they usually do not comment on them. The reason given was that comments are more likely to get you “attacked”. Another used expressed that they feel a “like” does not influence anything different in comparison to a comment (Dvir-Gvirsman 2025). There is no statistically significant correlation between the number of likes or the number of followers and comments (Saeidi and Baradari 2023). There also does not seem to be a correlation between the quality of a video and engagement (Benajiba et al. 2023). Another study found that health-related posts were more likely to be shared with other users but there was no correlation to a high amount of likes or comments (Tenenboim 2022).

We analysed the highly liked and commented posts shown in Figs. 12 and 13 as red dots. All results are shown below in Tables 6 and 7, respectively. Posts in both categories showed 67% misinformation. All posts in both categories were created by influencers. The length of the highly liked posts and highly commented posts varied substantially.

**Table 6:** Parameters of extraordinary highly liked posts

File number	Occupational group	Video length in sec	Ingredients	Source	Misinformation
20	Influencer	21	Estrogen/gestagen	Several	No
40	Influencer	171	Fentanyl, opioids, cocaine	None	Yes
83	Influencer, no expertise	100	Cannabis	None	Yes

**Table 7:** Parameters of extraordinary highly commented posts

File number	Occupational group	Video length in sec	Ingredients	Source	Misinformation
20	Influencer	21	Estrogen/gestagen	Several	No
26	Influencer	25	Cannabis	One	Yes
40	Influencer	171	Fentanyl, opioids, cocaine	None	Yes

### Successful content on social media

In our sample, 8 out of 10 most viewed posts were created by people without expertise with a range from 6,200,000 to 17,000,000 views (Table 2). Also, 8 out of 10 creators were categorised as “influencer” among other occupational groups. Four of these videos contained misinformation. All posts were created in English.

This finding is supported by other studies. Personal TikTok accounts observably use more digital effects and features for creating their content and as a result achieve high user engagement (McCashin and Murphy 2023). This was lacking in public health accounts leading to less attention (McCashin and Murphy 2023). Official public health accounts should consider investing more time in using the full range of creational possibilities on TikTok to design their content more appealing to users. Other studies suggest that unless professionals develop a deeper understanding of the app and its characteristics, they may miss important education opportunities especially concerning young users (McCashin and Murphy 2023). Results of other studies investigating health-related topics are shown in Table 8.

**Table 8:** Findings of other studies about TikTok’s influence on health topics

Source	Topic/focus	Method	Key findings
Baghdadi et al. (2023)	COVID-19 misinformation on TikTok	Content analysis of TikTok videos with #coronavirus	High engagement with COVID-19 misinformation; potential threat to public health behavior
Berg et al. (2023)	Social media policies on cannabis promotion	Review of platform policies	Loosely defined guidelines may promote cannabis marketing and underage exposure
Bozzola et al. (2022)	Social media use in children and adolescents	Scoping review	Identified risks like cyberbullying, addiction, and exposure to harmful content
Coates et al. (2019)	Influencer marketing and children’s food intake	Randomised trial with children	Unhealthy food promotion increased intake; healthy food promotion had no effect
Johnson et al. (2025)	Portrayal of prescription stimulants on TikTok	Systematic content analysis	Predominantly positive depictions may influence medication requests
McCashin and Murphy (2023)	TikTok and youth mental health	Systematic review and content analysis; descriptive table in Microsoft Excel	TikTok is under-researched, considering psychological topics, despite its popularity among youth; adverse effects on mental health are possible
Montag et al. (2021)	Psychology of TikTok use	Narrative review	TikTok’s design may lead to addictive behaviors and affect well-being
Olvera et al. (2021)	TikTok tics phenomenon	Descriptive and quantitative assessment of TikTok content	Surge in tic-like behaviors among adolescents linked to TikTok content
Pedrouzo and Krynski (2023)	Children and adolescents on social media	Narrative review	TikTok’s algorithm is highly addictive to children; excessive use may lead to adverse cognitive effects
Powell and Pring (2024)	Social media influencers and health outcomes	Systematic review	Influencers can significantly impact health behaviors and outcomes
Radin and Light (2022)	TikTok in science communication	Case study	TikTok presents opportunities for teaching and learning science online
Ranpariya et al. (2022)	Dermatology influencers on TikTok	Content analysis	Promotional content often lacks disclosure; potential misinformation; accounts focused on dermatology, lacking formal qualifications, demonstrated the greatest frequency of promotional material
Wu et al. (2022)	Cigar promotions on TikTok	Systematic content analysis	Influencers promote cigars without age restrictions; potential youth exposure
Zenone et al. (2021)	TikTok and public health research agenda	Commentary	Areas for future research on TikTok’s public health impact should include health-risk product marketing, portrayal of illness and medical advice, and (mis)information diffusion

## Limitations

Since only 121 posts were analysed, no general conclusions on the presentation of pharmacological content on TikTok can be drawn. Further research is needed to investigate whether the findings of the present study are generally applicable. Since this paper focused specifically on popular posts and the number of likes was filtered, less popular posts with different quality of information may not have been found. Thus, our findings may include selection bias. Additionally, the classification of misinformation was conducted based on today’s level of medical research and consensus. The category misinformation was analysed in binary outcomes. This may oversimplify our findings. In Table 4, we subcategorised misinformation, but future research should also focus on the nuances of misinformation on a deeper level so that findings are more specific.

The reliability of our findings may be reduced because of the initial classification of misinformation by one coder. Future studies should include inter-coder testing. In future studies, more extensive statistical analysis of data sets should be performed.

Because of different levels of popularity concerning the drugs/active ingredients, there were varying numbers of contributions to the individual groups. This causes a restricted comparability between the ingredients. Sometimes several drugs/active ingredients were mentioned per video, so the presence of misinformation cannot be clearly assigned to one individual ingredient. Aside from that, smaller groups of active substances were only mentioned in one post. These smaller groups are not representative. The numbers of views and likes were collected over a period of several weeks. Because of varying levels of fluctuations on TikTok, this might cause restricted comparability between the individual videos. One video was deleted while this research took place so the number of views could not be established.

This study did not investigate the impact of language and cultural differences on perception and creation of posts. Despite these limitations, our findings suggest a concerning trend of misinformation on pharmacological topics on TikTok.

## Conclusions

All results shown above indicate possibly dangerous consequences concerning health-related content on TikTok. The statistically significant association between occupational group and misinformation and source suggests that the professional background of creators may influence the accuracy of the information presented and whether a source of reference is used. With 32% of misinformation present, it is imperative to develop better control mechanisms for TikTok content, not only to prevent misinformation from spreading, but also to regulate advertisements. Guidelines concerning advertising on social media are often vague and are not rigorously followed (Berg et al. 2023). It is important to investigate TikTok independently from other social media platforms because the audience and content can be highly different to other social media even on the same profiles (Comp et al. 2021). For example, a TikTok profile with 1.1 million followers can simultaneously have less than 1000 followers on Instagram (Comp et al. 2021). Aside from that, TikTok creates a sense of community and belonging through its nature of sharing and commenting (Comp et al. 2021). The effects need to be observed especially for vulnerable users like children.

Other studies also found that health-related information on TikTok often does not meet scientifically established standards (Montag et al. 2021). This is especially concerning for young users since it is known that they are more likely to show risky behaviour online including substance abuse (Bozzola et al. 2022). With estrogen/gestagen, cocaine, and opioids being the most popular topics, it is clear where interests in mostly young people lie. Misinformation considering topics such as cannabis or other drugs may lead to a false understanding and trivialization of these substances. Especially for addicting drugs, content needs to be supervised and banned if needed. Major alcohol brands gained popularity on social media by advertising their products and evidence shows that viewers of this content normalize the behaviour presented (Bozzola et al. 2022). A correlation of social media use and addiction has already been established (Bozzola et al. 2022).

## Future studies

It is essential that future studies investigate other social media regarding the presentation of pharmacological content. Future studies should include broader sampling strategies and use TikTok Research API. Stricter guidelines for advertisement and quality control of information presented should be established. Considering the findings of this research, we suggest that TikTok use should not be allowed for people younger than 16 years. Children are highly vulnerable to being influenced by social media influencers and advertisements especially concerning junk food, mental health, and addiction behaviour (Pedrouzo and Krynski 2023). It is of high importance to establish control mechanisms when registering for use of TikTok, since many children do not use their real birth of date and therefore gain access to unsuitable content (Berg et al. 2023).

Health care practitioners must be made aware of the consequences of TikTok use and understand pharmacology-related topics and beliefs. Professionals should address this in patient-doctor conversations to achieve better education and understanding for the information discussed. Common misconceptions can possibly be corrected and insecurities arising because of content consumed on social media minimised. Parents must be made aware of the risks of TikTok use and guided to teaching their children safe social media use (Pedrouzo and Krynski 2023).

TikTok is still rarely accessed by institutional accounts (McCashin and Murphy 2023). This is also shown in our research since the majority of the content was created by accounts without expertise. It needs to be investigated in which way professionals can use social media platforms as an opportunity to convey pharmacology-related education and engage with the young population on TikTok.

## Data Availability

All source data for this study are available upon reasonable request.

## Abbreviations

CBD: Cannabidiol
Fig.: Figure
LSD: Lysergic acid diethylamide
MDMA: 3,4-Methylendioxy-N-methylamphetamine
SPRM: Selective progesterone receptor modulator
THC: Tetrahydrocannabinol
